# Subacute cortical infarct: the value of contrast-enhanced FLAIR images in inconclusive DWI

**DOI:** 10.1590/0100-3984.2017.0188

**Published:** 2019

**Authors:** Pantelis Kraniotis, Aikaterini Solomou

**Affiliations:** 1 University General Hospital of Patras, Patras, Greece.

**Figure 1:**
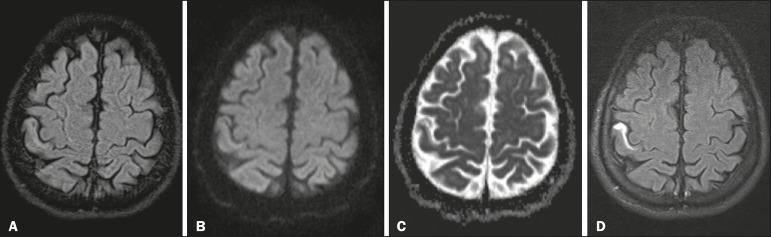
**A:** Axial FLAIR image. Note the subtly hyperintense signal in the anterior cortex of the postcentral gyrus. **B,C:** Corresponding axial DWI (**B**) and ADC map (**C**). There is a barely discernible hyperintense signal on DWI, although there is no evidence of low signal intensity on the ADC map (i.e., there is no restricted diffusion in the region). **D:** Gadolinium-enhanced FLAIR image showing marked contrast uptake in the affected area of the postcentral gyrus.

Dear Editor,

A 44-year-old patient presented with axial sensorimotor deficit, dating back approximately 10 days. The history was significant for diabetes, alcoholism, and cognitive impairment, making it difficult to assess the recent history and symptoms. The patient was submitted to brain magnetic resonance imaging (MRI) with T2-weighted imaging (T2WI), fluid-attenuated inversion recovery (FLAIR) sequences, susceptibility weighted imaging, and diffusion-weighted imaging (DWI), as well as T1-weighted imaging (T1WI), before and after intravenous gadolinium administration, in the axial, sagittal, and coronal planes. In the right postcentral gyrus, MRI revealed a cortical lesion, which showed a hyperintense signal on FLAIR image (Figure 1A). The lesion could be due to a new or older infarct. However, there was no restricted diffusion suggestive of a recent infarct (Figures 1B and 1C). Contrast-enhanced FLAIR imaging revealed marked cortical enhancement in the right postcentral gyrus, consistent with a subacute cortical infarct (Figure 1D).

Diabetes mellitus is a well-recognized risk factor for ischemic stroke, which is a leading cause of death and disability. MRI is quite sensitive in detecting ischemic changes. T2WI is more sensitive than is T1WI, and T1WI after gadolinium administration can provide valuable information for the accurate diagnosis^(^^1^^,^^2^^)^. Intravascular enhancement, although not specific, is considered a sign of ischemia on conventional MRI. Contrast enhancement in the central nervous system is the result of a combination of disruption of the blood-brain barrier, high vascularity, and contrast leakage into the lymphatic system^(^^3^^-^^6^^)^. After one week, infarcts show parenchymal enhancement, due to breakdown of the blood-brain barrier^(^^7^^)^.

New imaging techniques, such as DWI and perfusion-weighted imaging, have increased the accuracy of the diagnosis of acute cerebral ischemia, although there are some cases in which it cannot be distinguished from other entities^(^^8^^,^^9^^)^. In addition, because of pseudonormalization, subacute infarcts may not show restricted diffusion on DWI.

FLAIR is highly sensitive for the detection of ischemic lesions. Although it is considered to be heavily T2-weighted, rendering cerebrospinal fluid as dark, it also shows mild contrast enhancement on T1WI, which is responsible for the increased conspicuity of gadolinium enhancement. Pathologic conditions that present contrast enhancement on T1WI usually show marked enhancement on contrast-enhanced FLAIR^(^^10^^)^. This is exactly what occurred in the case presented here, in which DWI pseudonormalization did not help reveal the subacute cortical infarct. When a subacute cortical infarct is suspected, delayed contrast-enhanced FLAIR imaging is the best choice for demonstrating the lesion and for differentiating it from an older lesion with gliosis.
